# Trigeminal neuralgia caused by venous compression: a comprehensive literature review

**DOI:** 10.25122/jml-2024-0040

**Published:** 2024-05

**Authors:** Abdallah Alzeeralhouseini, Galina Moisak, Ekaterina Labzina, Jamil Rzaev

**Affiliations:** 1 Department of Neurosurgery, Novosibirsk State Medical University, Novosibirsk, Russia; 2 Department of Neurosurgery, Novosibirsk State University, Novosibirsk, Russia; 3 Department of Neurosurgery, Federal Center of Neurosurgery, Ministry of Health, Novosibirsk, Russia

**Keywords:** trigeminal neuralgia, microvascular decompression, venous compression, surgical outcomes, pain management

## Abstract

Trigeminal neuralgia (TN), a severe facial pain condition, is often treated with microvascular decompression (MVD). While MVD is effective for arterial neurovascular compression, its efficacy in cases of venous compression and the intraoperative management of such cases remain areas of debate. This review aimed to analyze the intraoperative management strategies for offending veins during MVD and evaluate the outcomes of these procedures in cases of TN with purely venous compression. An extensive review of studies reporting on the intraoperative handling of veins and the surgical outcomes of MVD in purely venous compression cases was conducted. Fifteen full-text studies were included, encompassing a total of 600 patients. Notably, 82.33% of these patients achieved a Barrow Neurological Institute (BNI) I pain score, with follow-up periods ranging from 3 months to 12 years. MVD is a viable and effective treatment option for TN in cases of venous compression, with a significant proportion of patients experiencing substantial pain relief.

## INTRODUCTION

Trigeminal Neuralgia (TN) is a debilitating disorder characterized by recurrent, unilateral, brief electric shock-like pains, abrupt in onset and termination, and is confined to the areas served by one or more divisions of the trigeminal nerve. These pains often occur in response to innocuous stimuli such as chewing, speaking, swallowing, or brushing teeth, severely impairing patients' quality of life [1-3]. Despite its relatively rare occurrence, with a prevalence ranging from 0.03 to 0.3% and an annual incidence rate of 4.7 per 100,000 individuals, TN is the most common form of cranial neuralgia [4]. The condition shows a female predominance, with a male-to-female ratio between 1:1.5 and 1:1.7, and is primarily diagnosed in individuals aged 50 to 60. The V2 and V3 branches of the trigeminal nerve are most commonly affected, with the ophthalmic (V1) branch being involved in isolation in less than 5% of cases. Moreover, 2–4% of patients with multiple sclerosis (MS) exhibit trigeminal symptoms, with MS present in 2–14% of individuals with TN [5-9].

TN can be classified based on its etiology. Burchiel *et al*. [10] distinguished idiopathic TN from trigeminal neuropathies or facial pain caused by other factors such as nerve injury, MS, or herpes zoster, further categorizing idiopathic TN into two types: TN1, characterized by sharp, shooting, electrical shock-like episodic pain, and TN2, which involves aching, throbbing, burning pain that is constant in more than 50% of cases.

Although arterial compression of the trigeminal nerve root at the root entry zone (TREZ) is a well-established cause of TN, neurovascular conflict involving venous compression is increasingly recognized as a significant contributing factor [11]. Treatment varies, with carbamazepine being the gold standard for drug therapy [12]. However, surgical intervention is required for some patients due to intractable symptoms or intolerance to medications. Surgical options include neurectomy, percutaneous ablation, and procedures aimed at relieving nerve compression along its path, such as microvascular decompression (MVD), which is recognized as the most effective treatment for classical TN associated with arterial compression of the nerve root. Long-term cure rates for MVD range from 70 to 90%, though in cases of TN caused by venous compression, the recurrence rate is believed to be higher [13-21].

Recent research into TN has explored various aspects of management and underlying pathophysiology. A study has examined the structural changes in cortical morphology in classical TN patients, suggesting possible pathophysiological changes underlying the disease [22]. Updated guidelines emphasize a multidisciplinary approach and evidence-based decision-making for diagnosis and treatment [23]. Additionally, research has explored new treatment avenues, including the potential of gabapentin as a safer and more effective medication [24], as well as the safety and efficacy of surgical interventions like Gamma Knife stereotactic radiosurgery for drug-resistant TN [25,26]. Additionally, the incidence and management of TN in specific populations, like those with multiple sclerosis, have also been investigated [27]. Other investigations have focused on non-invasive therapies like transcutaneous electrical nerve stimulation (TENS) [28], factors influencing surgical outcomes in microvascular decompression procedures [29], and the role of radiofrequency ablation (RFA) in pain management [30]. These studies collectively advance our understanding of TN and enhance patient care.

## MATERIAL AND METHODS

### Search strategy

**Figure 1:**
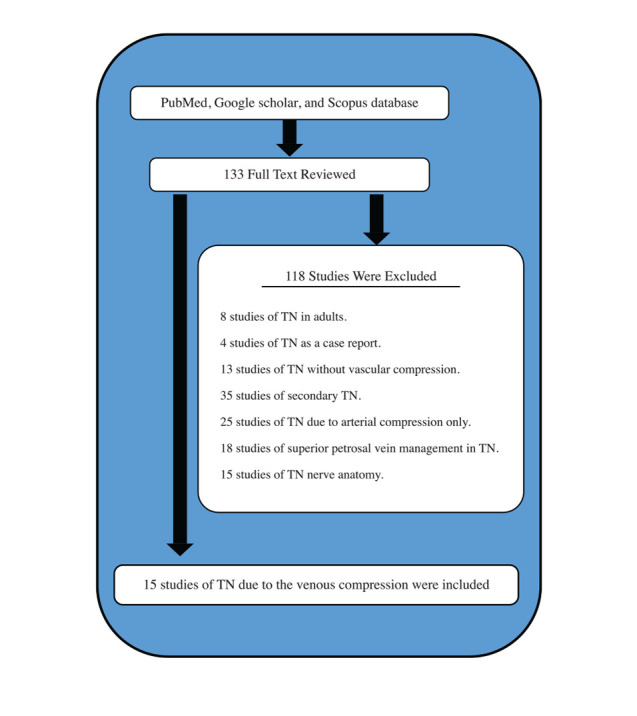
Study selection process

A comprehensive literature search was conducted using the following databases: PubMed, Google Scholar, and Scopus (Figure 1). The search was performed using a combination of medical subject headings (MeSH) and keywords, including 'trigeminal neuralgia', 'vascular compression syndrome', 'cranial nerve diseases', 'microvascular decompression', 'venous compression', 'neurosurgery', 'neurology', and 'review'. The search was limited to studies published between January 2004 and December 2022.

### Study selection

The studies were included if they met the following inclusion criteria: 1) original research articles, systematic reviews, and meta-analyses reporting on trigeminal neuralgia caused by venous compression, 2) studies that discussed the pathophysiology, diagnosis, management, and outcomes of this condition, 3) studies that provided a detailed description of the surgical techniques used in the treatment of trigeminal neuralgia caused by venous compression, and 4) studies that reported on the long-term outcomes of the surgical treatment. The studies were excluded if they were case reports, letters to the editor, or conference abstracts.

### Data extraction

Four independent reviewers screened the titles and abstracts of the articles retrieved from the search to identify potentially relevant studies. Next, full-text articles were obtained and thoroughly reviewed. A standardized data extraction form was used to collect information from the studies. The extracted data included study design, patient demographics, etiology of trigeminal neuralgia, diagnostic criteria, imaging modalities, surgical techniques, intraoperative findings, postoperative outcomes, and complications.

### Data synthesis

A narrative synthesis approach was used to summarize the data. The data were organized by the following categories: pathophysiology, diagnosis, management, surgical techniques, and outcomes. A descriptive analysis was performed to summarize the characteristics of the included studies. Meta-analysis was not performed due to the heterogeneity of the studies.

### Outcome measures

The Barrow Neurological Institute (BNI) pain intensity and facial numbness scores were the primary outcome measures for pain control and facial numbness, respectively. For studies that did not explicitly report BNI scores, the following equivalences were used: excellent was considered analogous to BNI I, good to BNI II, fair to BNI III, and poor to BNI IV/V.

### Potential bias

A rigorous approach was implemented to reduce bias in this systematic review. The study team used a dual-reviewer approach to identify, evaluate, and include relevant papers for impartial selection. This method allows many reviewers to evaluate papers independently, eliminating selection bias. Agreement or contact with a third reviewer resolved disagreements between reviewers, enhancing the objectivity of the research selection.

### Study assessment

We utilized the Cochrane Risk of Bias tool for randomized controlled trials (RCTs) and the Newcastle-Ottawa Scale for non-randomized studies to evaluate the methodological quality of the included studies. These techniques helped assess the methodological quality of the studies included, including randomization, blinding, outcome data completeness, and group comparability.

We employed the Cochrane method for RCTs to rigorously examine biases across multiple dimensions: selection, performance, detection, attrition, and reporting. Each study was classified as having a 'low', 'high', or 'unclear' risk of bias in these categories. This thorough analysis allowed us to identify studies with potential methodological shortcomings that could undermine the reliability of their findings. For non-randomized studies, the Newcastle-Ottawa Scale was used to assess study quality based on three broad categories: group selection, comparability, and ascertainment of the exposure or outcome of interest for case-control or cohort studies. A maximum of nine stars were given to studies in each category, indicating excellent quality. This scale enabled the research team to differentiate among studies of varying quality and to analyze how the quality of the studies influenced the review outcomes.

## RESULTS

### Study characteristics

After study selection, 15 full-text studies were included [31-45]. These studies collectively analyzed 600 patients, representing 11.4% of the total 5,271 patients studied over 18 years from 2004 to 2022, who experienced pure venous conflicts. Follow-up ranged between 3 months to 12 years across studies. The following information was independently extracted: first author, publication year, median patient age, gender, number of MVD surgeries, number of patients with venous compression, affected side, intraoperative management of the offending vein, length of study follow-up, long-term pain relief, and complications (Tables 1 and 2).

**Table 1:** Demographic characteristics of patients undergoing microvascular decompression for venous compression in TN

№	First author	Publication year	Median patient age	Gender	MVD (*n*)	Patients (*n*)	Venous compression (*n*)	Affected side
1	Matsushima *et al*.	2004	50	F: M 6:1	121	121	7 5.79%	Unilateral, NA №
2	Zhong *et al*.	2008	64.4	F:M 58 (unk g)	407	407	58 14.25%	NA №
3	Sekula *et al*.	2009	48.1	F 14	114	114	14 12.28%	R: L 6:8
4	Hong *et al*.	2011	55.6	F:M 14:1	343	343	15 4.37%	Unilateral, NA №
5	Li *et al*.	2014	55.7	F: M 13:2	231	231	15 6.49%	Unilateral, NA №
6	Feng *et al*.	2015	60.7	F:M 17:4	118	118	21 17.8%	R: L 10:11
7	Dumot *et al*.	2015	48.59	F: M 105(unk g)	326	326	105 doc (of 124) 32.21%	R: L 81:43
8	Shulev *et al*.	2016	60±12	F: M 21:19	421	421	40 9.5%	Unilateral, NA №
9	Inoue	2017	59	F:M 26:5	280	280	31 11.07%	R: L 22:9
10	Dumot *et al*.	2017	46.6	F: M 25:30	313	313	55 17.57%	R: L 41:14
11	Zhao *et al*.	2018	NA	F:M 18:16	369	369	34 9.21%	R: L 21:13
12	Wu *et al*.	2018	54± 9.6	F:M 41:23	1456	1456	64 4.4%	R: L 23:41
13	Kasuya *et al*.	2020	59.4	F:M 39:19	308	288	58 20.14%	R: L 36:22
14	Wang *et al*.	2021	56	F:M 20:14	318	222 doc	34 15.32%	R: L 24:10
15	Baldauf *et al*.	2022	58.4	F: M 34:15	262	262	49 18.7%	R: L 29:20
		2004- 2022	> 48	F: 288 (48 %) M:149 (24%) F:M 163 (27.2%) unk g	5387	5271	600 (11.4 %)	

2004-2022; F, female; M, male; R, right; L, left; doc, documented; NA, not available; NA №, not available number.

Unk g: the exact female: male ratio is unknown

**Table 2:** Vein management in neurovascular compression: long-term results and complications

№	First author	Intraoperative management of the offending vein	Length of study follow-up	Long-term pain relief	Complications
1	Matsushima *et al*.	Sacrificed – 4 pts Interposed – 3 pts	6 months – 15 years; median 7 years, 7 months	3 pts- Excellent 4 pts – Good	4 pts – Facial paresthesia
2	Zhong *et al*.	Sacrificed – 56 pts Saved – 2pts	NA	55 pts –Excellent 3 pts – Failed	3 pts – Ipsilateral cerebellar edema and brainstem shift later, they got facial numbness and hemiparesis in the contralateral side
3	Sekula *et al*.	Sacrificed – for small veins Transposed or interposed – for large veins	12–24 months; median 18.4 months	10 pts – Excellent 2 pts – Fair 2 pts – Poor	NA
4	Hong *et al*.	12 pts – Sacrificed 3 pts – Interposed	14 to 26 months; median 19 months	11 pts – Excellent 2 pts – Good 2 pts – Failed	5 pts – Facial numbness (2 pts with it before the operation)
5	Li *et al*.	15 pts- Interposed	10 to 20 months; median 14.5 months	12 pts – Excellent 1 p- Good 2 pts- Failed	3 pts – Facial numbness
6	Feng *et al*.	4 pts – Sacrificed 17 pts – Interposed	12 to 30 months; median 20 months	13 pts – Excellent 7 pts – Good 1 p – Failure	6 pts – Facial numbness
7	Dumot *et al*.	50 NVC – Sacrificed 86 NVC – transposed 12 pts – Sacrificed SPV 112 pts – saved SPV	1 to 9.4 years; median 4.7 years (only for 105 pts)	105 pts documented Follow-up on long-term 1– BNI I or II- 79 pts (75.2 %). 2 – in mixed group, relief was achieved in 67.4 % 3 – In the pure venous group, relief achieved in 77.3 %	5pts – Facial numbness 1p – Facial numbness and infarction
8	Shulev *et al*.	1^st^ group- 11pts. pure venous – Sacrificed 2^nd^ group – 29 pts. Arterial – Transposed and the venous- Scarified	NA	In the first group: BNI 1 –9 pts. BNI 2 –1 p. BNI >3 – 1 p. In the second group: BNI 1 – 20 pts. BNI 2 – 3 pts. BNI >3 – 6 pts.	9 pts – Facial numbness in both groups
9	Inoue *et al*.	20 pts – Scarified 11 pts – Transposed	12-95 months; median 33 months	BNI1 1 – 31 pts	7 pts – Facial numbness
10	Dumot *et al*.	The conflicting artery in mixed conflict – transposed and the compressive vein(s) scarified. If cleavage was not achieved, then the vein was scarified	1 - 9.37 years; Median 4.9 years	BNI 1 – 34 pts. BNI 2 – 5 pts. BNI >3 – 16 pts.	1p – Cerebellar infarction 1p – Cerebellar infarction & trochlear palsy 1p – Facial weakness 2 pts – Thromboembolic event
11	Zhao *et al*.	34 pts – Interposed	12-71 months; Median 50.5 months	26 pts – Excellent 4 pts – Good 3 pts – Fair 1 p – Unimproved	2 pts – CSF leakage
12	Wu *et al*.	Interposing for Small <1 mm and medium 1-3 mm sized veins and transposing of the large veins > 3 mm	3 to 60 months; median (4.3±12.0) months	51 pts – Excellent 6 pts – Good 6 pts – Fair 1 p – Poor	10 pts – Facial numbness, 1 p – Intracranial infection 1 p – Hearing loss.
13	Kasuya *et al*.	44 pts – scarified 14 pts –transposed	5 to 139 months; (mean, 55 ± 38 months, median 48 months).	BNI I-2 = 49 pts. BNI 3-5= 9 pts.	14 pts – Venous infarction of the middle cerebellar peduncle. 8 pts – Facial numbness
14	Wang *et al*.	Scarifying small veins, and transposing or interposing large veins	32–92 months; median 45 months	BNI 1 – 23 pts BNI>2 – 11 pts Delayed – 8 pts Recurrence – 1p	6 pts – Complications were documented
15	Baldauf *et al*.	17 pts – Scarified 32 pts – Transposed	3–144 months; median 42.1 months	BNI 1 – 32 pts. BNI 2 – 3 pts. BNI >3 – 14 pts	14 pts – Facial numbers, 4 pts – Transient partial hearing loss, dizziness, meningitis, and transient double vision

P, Patient; pts, patients; Scarified, Coagulation and Cut off; NVC, Neurovascular conflict; SPV, Superior Petrosal Vein; BNI, Barrow Neurological Institute; NA, Not Available; CSF, Cerebrospinal fluid

### Pain control and facial numbness rates

Fifteen studies reported pain control and facial numbness rates following MVD in a total of 600 patients with venous compression-related trigeminal neuralgia. Detailed results are presented in Tables 3 and 4.

**Table 3:** Barrow Neurological Institute (BNI) pain intensity score

1	BNI I–II	494 (82.33%)
2	BNI III–V	106 (17.66%)

**Table 4:** Evaluation of numbness by BNI facial numbness score

1	BNI I	518 (86.34%)
2	BNI II-IV	82 (13.66%)

### Intraoperative management of venous compression

Intraoperative treatment for venous compression was performed in all 600 patients across the 15 included studies. Due to incomplete reporting of patient numbers in some studies, the specific tactics employed for venous decompression were categorized as follows: transposition with Teflon use, transposition with fibrin glue, interposition, or dissection of the offending vein without additional manipulation. Internal neurolysis or nerve squeezing were not considered primary treatment tactics for venous compression in this review, except in cases of “impossible sufficient decompression”, as reported by Wu *et al*. [42].

The decision to perform surgical intervention (coagulation and cut-off) for trigeminal nerve decompression in the context of venous compression is not a uniform procedure but is instead adapted based on a series of critical assessments. These include the diameter of the vein involved, specifically emphasizing diameters less than 2 mm [44,45] and less than 1 mm [36], which are significant indicators for employing coagulation and the subsequent cut-off tactic. Moreover, the anatomical relevance of the compressed vein, especially if it constitutes one of the tributaries of the superior petrosal vein (SPV) trunks [31,37], plays a crucial role in the decision to proceed with this method. The outcomes of occlusion tests, particularly negative results [32], further justify the intervention, alongside comparisons of the diameter of the vein with other vital anatomical structures such as the superior cerebellar artery (SCA) [33] and the vein of the cerebellopontine fissure (VCPF) [39]. In situations when cleaving the vein away is not feasible without risking adverse outcomes, coagulation followed by cutting off the vein emerged as the chosen method of action [34, 40, 43].

This comprehensive review of surgical strategies for trigeminal neuralgia caused by venous compression emphasizes the importance of personalized treatment. By incorporating specific criteria and methodologies from relevant research [31-34, 36-40, 43-45], this manuscript aligns closely with established clinical guidelines, highlighting the need for informed decision-making to ensure the safety and efficacy of procedures like coagulation and cut-off. This approach prioritizes minimizing the potential impact on cerebral venous drainage while effectively decompressing the trigeminal nerve.

### Female predominance in TN cases

The systematic review of 15 studies encompassing 600 cases of trigeminal neuralgia revealed a significant female predominance, with a female-to-male ratio of 1.5:1. This finding aligns with previous research demonstrating a higher incidence of TN in women. Most instances were detected among older individuals, mostly between 50 and 60. The prevalence of women with TN suggests that gender-specific biochemical, hormonal, or genetic variables may contribute to its development and presentation.

## DISCUSSION

The study provides a comprehensive review of outcomes and pain recurrence after MVD for TN caused solely by venous compression. The findings highlight the importance of considering venous compression at the trigeminal root entry zone (REZ), mid-cisternal segment, and petrous segment as potential causes of TN. Additionally, the study emphasizes that MVD can be an effective treatment option when the anatomy and mechanism of the offending vein are well understood. This systematic review provides important insights into the surgical management of patients with pure venous conflicts. Of 5271 patients, 600 (11.4%) had pure venous conflict, and the follow-up period ranged from 3 months to 12 years. These studies focused on the diagnostic methods, intra-operative management of the offending vein and their relation to the outcomes, length of study follow-up, long-term pain relief, and complications.

Venous compression makes the coagulation and cut-off method a crucial option in surgical therapy for TN, balancing decompression with venous drainage changes. While helpful for minor problematic veins, this method needs thorough preoperative and surgical assessment to reduce hazards. This strategy emphasizes the need for a personalized approach, anatomical accuracy, and the surgeon's carefulness in optimizing patient results.

Various authors have reported that preoperatively, the neurovascular conflict involving the trigeminal nerve root was confirmed through a combination of clinical evaluation and different MRI sequences, such as MRI 3D-TOF [34, 36, 37, 39, 40], CISS [45], FIESTA [39], SPGR-T1 with contrast [39], T1 with gadolinium [33, 37, 40], and 3D-T2 [37, 40]. Intraoperatively, during microvascular decompression surgery, the offending vein typically identified as the compressive factor on the trigeminal nerve root is most commonly located in the REZ zone [34, 36, 38, 45] and mid-cisternal zone [37, 39,41,44]. Across multiple studies, the transverse pontine vein emerged as the predominant offending vein in these cases [31, 36, 37,38].

Surgeons employed three main surgical techniques to decompress the offending vein: coagulation and cut-off (Co&Cu), interposition, and transposition [31-45]. Co&Cu, the most commonly used technique, was favored for various scenarios, including small veins (<2 mm or <1 mm), veins involved in the superior petrosal vein trunks, cases with negative occlusion tests, veins smaller than the superior cerebellar artery (SCA) or the vein of the cerebellopontine fissure, and situations where cleaving the vein was not feasible [31-34, 36, 37, 39, 40, 43-45]. Interposition and transposition were preferred for larger veins (>2 mm or >3 mm), medium-sized veins (1–2 mm), veins larger than the SCA or VCPF, cases with positive occlusion tests, or when coagulation was considered risky due to the size or location of the vein [25, 31-34, 37-39,41,43,45]. In addition, one study reported a unique technique involving sharp dissection and cauterization of the arachnoid and petrosal vein tributaries, followed by Teflon placement and band removal to ensure nerve mobility [35].

Co&Cu was the most commonly used technique for managing the offending vein. This technique involves coagulating and cutting the offending vein to prevent it from compressing the trigeminal nerve. Displacement of the offending vein was used in a few cases where Co&Cu was not feasible or effective. Long-term pain relief was reported in all 15 studies (Table 2). The follow-up duration ranged from 3 months to 12 years, and the long-term pain relief rate varied among studies, with rates ranging from 55% to 100%. For the studies that did not report BNI outcome scores, excellent was considered analogous to BNI I, good to BNI II, fair to BNI III, and poor to BNI IV/V. These studies defined the long-term pain relief rate as the percentage of patients with a BNI score of I–II (Table 2). The differences in pain relief rates among the studies may be due to patient selection, surgical technique, and follow-up duration variations. Complications were reported in all 15 studies (Table 2). The most common complications were facial numbness or hypoesthesia (BNI II-IV = 13.66%), which occurred in 11 studies. Other complications reported included hearing loss, tinnitus, cerebellar hematoma, and cerebrospinal fluid (CSF) leak. The rate of complications varied among the studies, ranging from 0% to 16.7%. However, the differences in complication rates among the studies may also be due to patient selection and surgical technique variations. Most patients experienced excellent and good pain relief (BNI I–II = 82.33%).

Finally, the location of neurovascular conflict (NVC) and the specific surgical techniques employed for vein management did not influence the outcomes related to pain improvement or resolution. No distinct anatomical features were found that could predict the success of treatment. Furthermore, the particular surgical procedures used did not affect the overall results. Additionally, further research is necessary for patients experiencing venous compression to identify the most effective surgical approach for managing the offending vein to ensure better outcomes, including less recurrence of pain and fewer complications.

### Female predominance in trigeminal neuralgia: implications

The observed female predominance among patients with TN offers insights into its epidemiology and maybe pathogenesis. This gender disparity has been linked to hormonal influences, differences in pain perception and reporting between genders, and genetic susceptibility. Notably, estrogen and progesterone receptors are present in the trigeminal ganglion, suggesting that hormonal fluctuations could heighten the sensitivity of trigeminal nerve fibers. This hormonal sensitivity could potentially exacerbate TN severity or prevalence in women, particularly during menopause.

Given the substantial proportion of female patients in TN cohorts, it is vital to consider the potential impact of gender on treatment outcomes, especially for procedures like MVD that target venous compression. Future studies should examine gender-specific variations in the effectiveness of MVD or other TN treatments, as the included studies did not consistently provide gender-specific results. Understanding these variations is essential for developing personalized treatment approaches that account for gender as a potential modifier of treatment response and prognosis.

### Comparison of study findings with the literature

The research indicated that coagulation and cut-off (Co&Cu) was the most common vein management method, with displacement used in less feasible circumstances. All trials demonstrated long-term pain alleviation, with success rates ranging from 55% to 100% depending on patient selection, surgical method, and follow-up time. The investigations showed different complications, including face numbness and hypoesthesia, emphasizing surgical accuracy and patient-specific care. A classification system for superior petrosal veins (SPVs) was created to stress the importance of keeping the cerebellopontine fissure vein open during surgery [46].

Additionally, fissurectomy relieved pain quickly and had high success rates for idiopathic non-infected fissures [47], while alcohol-blocking of the trigeminal nerve offered long-term relief for cases of medically intractable typical trigeminal neuralgia [48].

The review found no correlation between the neurovascular conflict (NVC) location among patients with TN and the specific surgical technique chosen for vein management. This suggests that anatomical variations in NVC location may not significantly influence pain relief outcomes [49]. Consequently, there is a need to refine and optimize surgical methods for addressing venous compression in patients with TN to maximize pain relief and minimize potential complications.

Research on neurovascular conflict highlighted the need to remove the petrosal vein from the nerve root to relieve pain [50]. Another study emphasized the need for a systematic approach to categorize and apply surgical techniques based on the specific location of venous compression-induced NVC in patients with TN [51]. This highlights the complexity of surgical decision-making when addressing TN related to venous compression and underscores the necessity for personalized treatment strategies to improve patient outcomes.

Percutaneous balloon compression for TN was shown to be beneficial regardless of gender, treatment type, or previous surgery [52]. These results imply that percutaneous balloon compression may be a successful therapy for patients with TN, highlighting the necessity for varied surgical approaches to venous compression.

The research found a high female incidence of TN, perhaps due to hormonal, pain perception, and genetic variables [53]. This gender gap necessitates more research into gender-specific therapy efficacy, particularly in MVD, to create personalized therapeutic methods that account for gender in treatment response and prognosis [54].

A recent study confirms the role of venous compression in TN pathogenesis and MVD efficacy [55]. Previous studies have stressed the necessity of accurate preoperative diagnoses and customized intraoperative techniques to improve outcomes and reduce complications. However, this analysis highlights the need for greater research into gender variations in treatment results, an issue that has been understudied.

The efficacy and safety of MVD underscores its importance in treating trigeminal neuralgia [56]. Demographic studies also underline the need for a multidisciplinary team of primary care doctors, dentists, neurologists, anesthesiologists, and neurosurgeons to effectively diagnose and treat TN [57]. These findings emphasize the complexity of factors such as gender, vascular compression, and treatment outcomes in TN, pointing to the need for personalized, multidisciplinary approaches to ensure optimal patient care and treatment efficacy. This comprehensive study strengthens the effectiveness of MVD for TN with pure venous compression and calls for sophisticated patient management and gender-specific outcome studies.

### Novelty

The novelty of this study is that it focuses on venous compression as a distinct etiology of trigeminal neuralgia, an area that remains relatively under-investigated and debated within the neurosurgical field. This study adds to the body of research by collecting and analyzing a large, detailed database that only contains cases of TN caused by venous compression. Historically, this patient group has been less studied compared to those with arterial compression-induced TN, and their treatment outcomes have proven more variable and challenging to predict. Thus, this study brought into focus, with specificity to this subgroup of patients, the intraoperative difficulties and decision-making processes related to venous compression and offered detailed insight into the efficacy and outcomes of several surgical approaches used in MVD.

Furthermore, this review makes a unique contribution to the literature by performing a quantitative synthesis of data from numerous studies, summarizing the long-term outcomes of different intraoperative management techniques in patients with purely venous conflicts. Our findings, which include detailed patient demographics, surgical techniques, and follow-up data, add substantial value to the existing literature and lay a foundation for future research. This study is innovative because it closely examines the surgical treatment of problematic veins and compares different methods to determine the most effective approaches for treating TN caused by venous compression. This deeper understanding of the anatomical and physiological nuances of venous compression could lead to new surgical practices that improve patient outcomes.

Given the complexity of TN from venous compression, this study suggests a more individualized neurovascular decompression surgery that carefully considers each patient's anatomy to achieve successful decompression and pain relief. These findings emphasize that further innovation in surgical techniques and patient management is possible, pointing towards a future where treatment for venous compression-induced TN is effective and nuanced.

In summary, the novelty of this study is represented by focused research on a relatively unexplored cause of trigeminal neuralgia, hence contributing to an in-depth understanding and awareness of the surgical outcomes related to venous compression, along with future approaches to the enhancement of patient care through surgery. This research addresses a specific and complex question in TN treatment, filling an important gap in the scientific literature and serving as a foundation for future innovative research standards.

## CONCLUSION

The management of compression veins has been a contentious topic, with some authors arguing that obstructing access or the presence of offending veins can lead to brainstem function issues or increased intracranial pressure, while others advocate for the preservation of these vessels to avoid potentially fatal outcomes. However, preserving the same vessel can lead to unsatisfactory decompression or pain recurrences. There is no accepted theory to guide vessel manipulation during MVD procedures. Most surgeons believe that understanding the normal anatomy of superior petrosal veins and their relationship with the trigeminal nerve is crucial for effective decompression. There is no consensus on the best approach for vessel manipulation during MVD, and further research is needed to determine optimal surgical techniques for managing offending veins to ensure excellent outcomes and minimize pain recurrence and complications.
